# The phytochemical profiling, pharmacological activities, and safety of *malva sylvestris*: a review

**DOI:** 10.1007/s00210-022-02329-w

**Published:** 2022-11-22

**Authors:** Gaber El-Saber Batiha, Stephano Tambo Tene, John Oluwafemi Teibo, Hazem M. Shaheen, Oyerinde Samson Oluwatoba, Titilade Kehinde Ayandeyi Teibo, Hayder M. Al-kuraishy, Ali I. Al-Garbee, Athanasios Alexiou, Marios Papadakis

**Affiliations:** 1 Department of Pharmacology and Therapeutics, Faculty of Veterinary Medicine, Damanhour University, Damanhour, 22511 AlBeheira Egypt; 2 Research Unit of Biochemistry of Medicinal Plants, Food Sciences and Nutrition, Department of Biochemistry, Faculty of Science, University of Dschang, Dschang, Cameroon; 3 Department of Biochemistry and Immunology, Ribeirão Preto Medical School, University of São Paulo, Ribeirão Preto, São Paulo Brazil; 4 Department of Veterinary Pharmacology and Toxicology, Faculty of Veterinary Medicine, University of Ilorin, Ilorin, Nigeria; 5 Department of Maternal-Infant and Public Health Nursing, College of Nursing, Ribeirão Preto, University of São Paulo, Ribeirão Preto, São Paulo Brazil; 6 Department of Clinical Pharmacology and Therapeutic Medicine, College of Medicine, Almustansiriyiah University, Bagh-Dad, Iraq; 7 Department of Science and Engineering, Novel Global Community Educational Foundation, Hebersham, NSW 2770 Australia; 8 AFNP Med, 1030 Wien, Austria; 9 Department of Surgery II, University Hospital Witten-Herdecke, University of Witten-Herdecke, Heusnerstrasse 40, 42283 Wuppertal, Germany

**Keywords:** *Malva sylvestris*, Traditional medicine, Phytochemistry profiling, Pharmacological activities, Safety

## Abstract

*Malva sylvestris* is a plant commonly found in Europe, Asia, and Africa. The leaves and flowers of this plant have been used for centuries in traditional medicine to treat various ailments such as cough, cold, diarrhoea, and constipation. Google Scholar, PubMed, Scopus, and Web of Science were used to search for relevant material on the phytochemical profiling and pharmacologic activities of *Malva sylvestris*. The techniques used in phytochemical profiling and the pharmacologic activity of each compound were extracted from the included studies, including in vitro, in vivo, and clinical studies. The phytochemical analysis of *Malva sylvestris* revealed that the leaves and flowers are the most commonly used parts of the plant and contain various bioactive compounds such as flavonoids, mucilages, terpenoids, phenol derivatives, coumarins, sterols, tannins, saponins, and alkaloids. These phytochemicals are responsible for the many pharmacological activities of *Malva sylvestris,* such as anti-inflammatory, antimicrobial, hepatoprotective, laxative, antiproliferative and antioxidant properties. This review has presented an overview of the antinociceptive and anti-inflammatory activities and the cytotoxic effects of *Malva sylvestris* on different types of cancer cells. It has also summarised the work on developing copper oxide nanoparticles using *Malva sylvestris* leaf extract and its potential use in food and medicine. This review aims to highlight the traditional uses, phytochemistry, pharmacological activities, and safety of *Malva sylvestris*.

## Introduction

*Malva sylvestris* L. is a flowering plant belonging to the Malvaceae family. In Europe, it is referred to as common mellow, and vilayatti or gulkhaira, kangani in India and Pakistan (Mustafa and Ali 2011), *Malva* in Portugal, Marva in Italy, ebegümeci in Turkey, and tole or khabazi in Iran. *Malva sylvestris* L. come from North Africa, Southwest Asia, and South Europe, although it is found worldwide as a weed. It prefers to grow in moist areas near oceans, swamps, canals, waterways, and meadows, among other spots (Razavi et al. 2011).

It may be an annual or a perennial plant and can reach a maximum height of around one meter. The blooms are brilliant pink with purple lines, and the leaves are heart-shaped with 5 to 7 lobes. *Malva sylvestris* is a rich source of phytochemical compounds (Nehir and Karakaya, 2004; Zhen-Yu 2005; Sabir and Rocha 2008; Tabaraki et al. 2012), and it has numerous health benefits against a variety of diseases (Jan et al. 2009; Pirbalouti et al. 2009; Razavi et al. 2011; Marouane et al. 2011), including diabetes mellitus and an autoimmune disorder (Akash et al. 2012, 2013). Traditionally, the plant has been used to treat various ailments, such as coughs, colds, diarrhoea, dysentery, hypertension, and skin diseases (Razavi et al. 2011). In recent years, there has been a resurgence of interest in studying natural products due to the increasing incidence of drug-resistant strains of bacteria and the side effects of synthetic drugs (Newman and Cragg 2016). This has led to a need for new therapeutic agents that are safe and effective. As a result, there has been a renewed interest in the study of plants with medicinal properties.

The phytochemistry and pharmacological activities of Malva sylvestris have been extensively investigated and reviewed. The purpose of this paper is to review the most recent data on the chemical composition and pharmacological activities of Malva sylvestris.

## Review of literature

A cooked leaf of *Malva sylvestris* may be eaten as a healthy vegetable in soups or simmered in yoghurt as a side dish to treat these ailments (Akash et al. 2012). However, it is also possible to cure these diseases with synthetic medications, which may have adverse effects. According to the author, a decoction made from the plant’s aerial parts is commonly utilised in traditional Persian medicine for treating gastrointestinal lesions, as a tonic for the gastrointestinal system, or even as a side dish. This is mainly due to the plant’s capacity to clean the colon (Rahimi et al. 2010). Besides, several preparations of *Malva sylvestris* (Mallow) flowers, leaves, and fruits, as well as their oral dosage forms, functional foods, and rectal douche, were investigated for their potential role in the treatment of stomach and rectal ulceration, and also pain and inflammation. in several Persian traditional and medical literature such as Qarabadin-e-azam (a lithograph book written by Hakim Tohfat ol Moemenin (Tonekaboni, 1670 AD), Azamkhan in 1853 AD), Qarabadin-e-salehi (Heravi, 1765), Qarabadin-e-kabir (Aghili Shirazi, 1772), and Qarabadin-e-kabir (Zargaran et al. 2014).

Almost all extracts of *Malva sylvestris* have been revealed to have anti-inflammatory, antioxidant, wound-healing (Prudente et al. 2013a; Sleiman and Daher 2009) and immunomodulatory properties (Pirbalouti et al. 2010). As previously described, *Malva sylvestris* (*Malvaceae*) grows yearly and may be found mainly in South-West Asia, North Africa, and Europe, among other places. Several chemicals with biological activities, including polysaccharides, carotenoids, polyphenols, fatty acids, ascorbic acid, and tocopherol, have been extracted from the edible sections of the plant in various concentrations (Cutillo et al. 2006; Pirbalouti and Koohpyeh 2011). Studies on this plant’s phytochemistry have reported the existence of many compounds with biological properties, including tannins, polysaccharides, flavonols, essential oil, anthocyanidins, flavones, anthocyanins, mucilagen, leucoanthocyanidines, coumarins, and terpenoids such as diterpenes, sesquiterpenes, and monoterpenes (Cutillo et al. 2006; Farina et al. 1995; Schulz et al. 2003; Gasparetto et al. 2012b).

Experts like Schulz et al. (2003) and Guarrera (2005) have revealed that *Malva sylvestris* has been used in traditional medicine for emollient and laxative functions since the dawn of time in popular treatment for its anti-inflammatory effects. Similarly, Guarrera (2005) found that *M. sylvestris* has antinociceptive, anti-inflammatory, and anti-carrageenan-induced paw oedema when locally administered In animal models (Gasparetto et al. 2012b). In addition to the antioxidant and antiradical characteristics of *M. sylvestris*, which have since been established by DellaGreca et al. (2009), the widespread use of this plant as an emollient, particularly its anti-inflammatory properties on the skin, has not piqued research interest. Besides being used as a natural product, *M. sylvestris* is also employed in infant feeding owing to its high micronutrients and other essential components (Guarrera 2003). Young leaves of the plant are often eaten raw in salads, while the shoots and older leaves are typically cooked and used as greens in soups and other dishes. Children, shepherds, and hunters eat immature fruits by sucking or chewing them (Barros et al. 2010; Neves et al. 2009).

All living things are protected from the outside world by a complex organ called skin, which performs a variety of vital functions such as defence against physical, chemical, and biological attacks that can lead to varying inflammatory conditions that manifest themselves on the skin's surface (Kanitakis 2002; Murphy et al. 2000). Keratinocytes (KC) predominate in this region of the body and are capable of producing cytokines, chemokines, and growth regulators (Uchi et al. 2000); however, many of these components are either retained in the cytoplasm or are not synthesised, which results in KC destruction and skin inflammation due to the damage (De Benedictis et al. 2001). In addition to inflammation, the skin can be affected by psoriasis, an immunological disorder, and atopic dermatitis, a persistent, recurrent skin conditions disease characterised by pruritic and eczematoid lesions. Both conditions can cause the skin to become inflamed (Gottlieb 2005; Leung et al. 2003).

Uchi et al. (2000) reveal that cytokines generated by Keranocytes are essential in controlling immunological and inflammatory reactions via their receptors on Langerhans cells, endothelial cells and dermal fibroblasts, Keratinocytes, and infiltrating T-cells. Contrarily, the adverse effects reported in patients with psoriasis or those with atopic dermatitis are characterised by dense infiltration of neutrophils, T cells, macrophages, dendritic cells, and natural killer cells (NK cells) (Clark and Kupper 2006), eosinophilia, and increased concentrations of interleukins (IL), prostaglandin (PG) E2, and immunoglobulin (Ig) E (Leung and Soter 2001).

Glucocorticoids and immunosuppressants, which are medications that have been used in conventional medicine to treat psoriasis and atopic dermatitis, have revealed significant side effects and toxicity, thus opening up the field of research and development of drugs with potent activity against the pathogens responsible for these pathologies but also with attenuated side effects (Leung et al. 2003, 2004). One of the solutions to these issues is topical medications, which are commonly used because they are more universally accepted than systemic medications and less evasive than systemic medications (Mrowietz and Reich 2009). Combining local treatment with systemic medication to treat skin problems may also be beneficial. Urpe et al. (2005) revealed the importance of the use of certain compounds such as tar, keratolytic, emollients, corticosteroids, dithranol, and vitamin D derivatives in topical applications to improve scaling and inflammation, as well as to minimise the pool of growth factors and cytokines, that also play an essential role in promoting inflammatory responses and hyperproliferation of the epidermis. These topical medications, when used regularly, are very important since they stimulate and enhance the mental attitude toward conditions such as psoriasis (Mason et al. 2000).

Diseases connected to metabolic changes, such as diabetes mellitus, that impact several different plants now control the body. This is in addition to the disorders stated before (Akash et al. 2011; Romila et al. 2010; Patel et al. 2012; Ponnusamy et al. 2011; Arif et al. 2014). In this regard, Pirbalouti et al. (2010) and Pirbalouti et al. (2012) recently reported a healing capacity of *Malva sylvestris* in alloxan-induced diabetic rats. Similarly, several studies have also demonstrated the antiulcerogenic and antiinflammatory properties of *Malva sylvestris* extracts (Conforti et al. 2008b; Sleiman and Daher 2009; Pirbalouti et al. 2010; Gasparetto et al. 2012b). Known as a plant with an antioxidant effect, several preliminary unpublished works have also reported that *Malva sylvestris* is endowed with hepatoprotective properties.

In India, *Malva sylvestris* grass is commonly known as mallow and can grow to a height of 4 ft. The stalkless flowers and leaves of *Malva sylvestris* are the most frequently used parts of traditional medicine to treat ailments, probably based on their high concentration of biologically active compounds. Tomoda et al. (1989) and Barros et al. (2010) report, respectively, that the active ingredients present in *Malva sylvestris* include mucilage, tannins, malvyn, malvidin, and the plant leaves are also a good source of nutraceuticals, including antioxidants (tocopherols, flavonoids, phenols, and carotenoids), unsaturated fatty acids (e.g. α-linolenic acid), and minerals. Numerous studies have shown that this plant has antimicrobial, anti-inflammatory, and antioxidant properties (Billeter et al. 1991; Pirbalouti et al. 2010).

The plant is a member of *Equisetopsida* class, *Magnoliidae* subclass, *Rosanae* superorder, *Malvales* order, *Malvaceae* family and *Malva* genus (Barros et al. 2010; Vandebroek et al. 2008; Garden 2010). When chewed, the flowers of *M. sylvestris* have a taste that is similar to that of mucilage, and they have practically no smell. The flowers are about 3–5 cm in width and feature an epicalyx on the end, with a length of no more than 20 mm for the rest of the stalk (Pljevljakušić et al. 2018). Their flowers are mainly composed of an epicalyx joined to elliptic-lanceolate sections positioned just below the calyx. These segments are shorter than the calyx. This part (calyx) comprises gamosepalous at the bases and five pubescent triangular lobes. Another component is a corolla that is 3 to 4 times the length of the calyx and has 5 wedge-shaped, serrated petals that are united to the stamen tube at the base.

Under the objective lens, many stamens, known as filaments, assemble into a stamina tube enclosed by tiny star-shaped and sparse, simple trichomes. In addition, numerous rumpled carpels, orbicular or often pubescent and enveloped in the stamen tube, are organised circularly around a core style that wraps up with multiple threadlike stigmas. Arayne et al. (2005) and Bonfill et al. (2006) reported that the epicalyx represents the main 3 to 7 partite in various cultivated varieties of *M. sylvestris*, the calyx represents the main 5 to 8 partite, and the corolla represents the main 5 to 10 partite in different cultivated varieties of *M. sylvestris*.

**Fig. 1:**
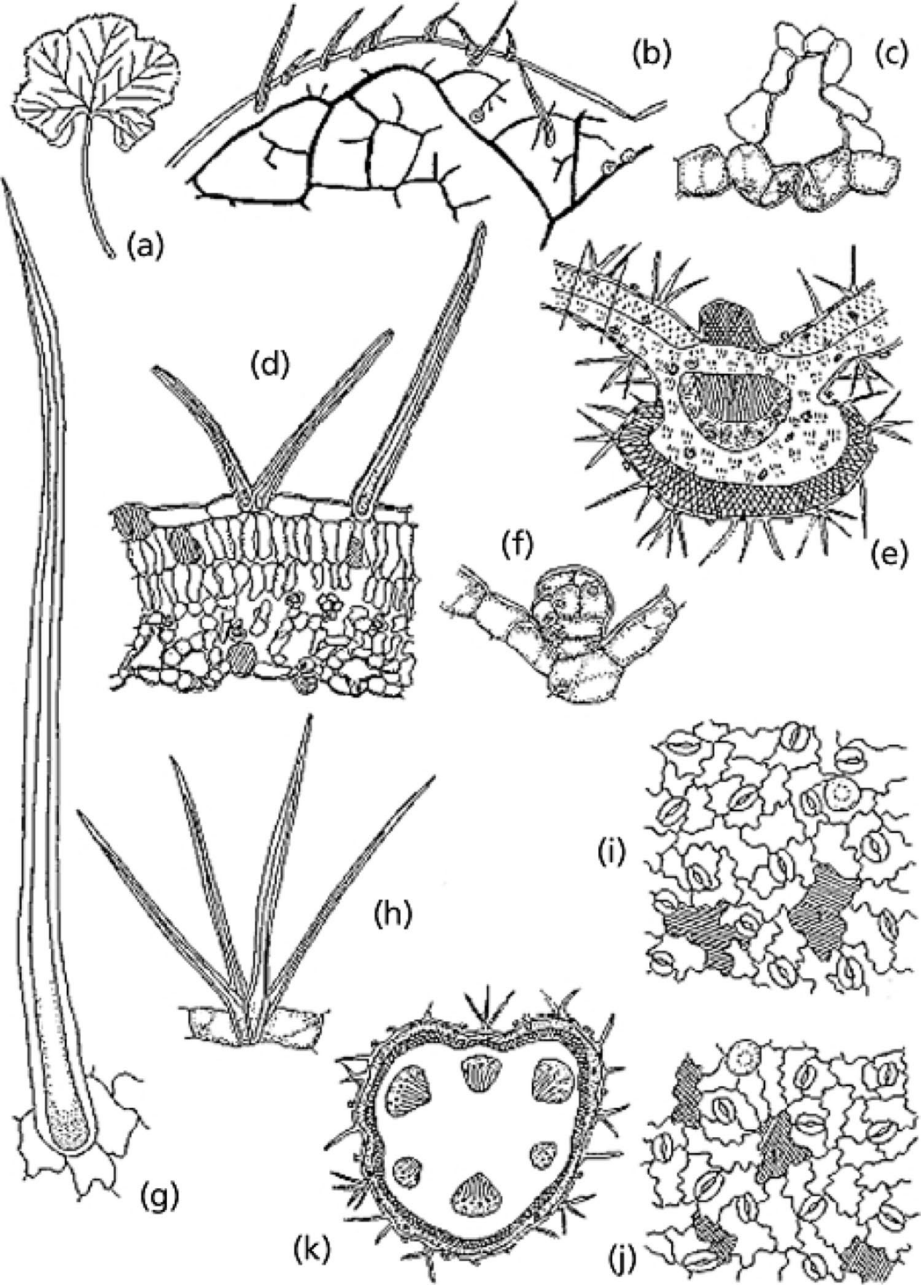
A leaf from *Malva sylvestris* L., *Malvaceae*, with morphoanatomical features. (**a**) Overall aspects; (**b**) leaf architectural style with perfect areole and polygonal; (**c**) stomatal detail; (**d**) depiction of the mesophyll with non-glandular trichomes; (**e**) the overall aspects of the midrib; (**f**) glandular trichome; (**g**) non-glandular trichome; (**h**) stellar trichome; (**i**, **j**) epidermal cells of the adaxial and (**k**) overall aspect of the petiole (Batistuzzo et al. 2002)

**Fig. 2:**
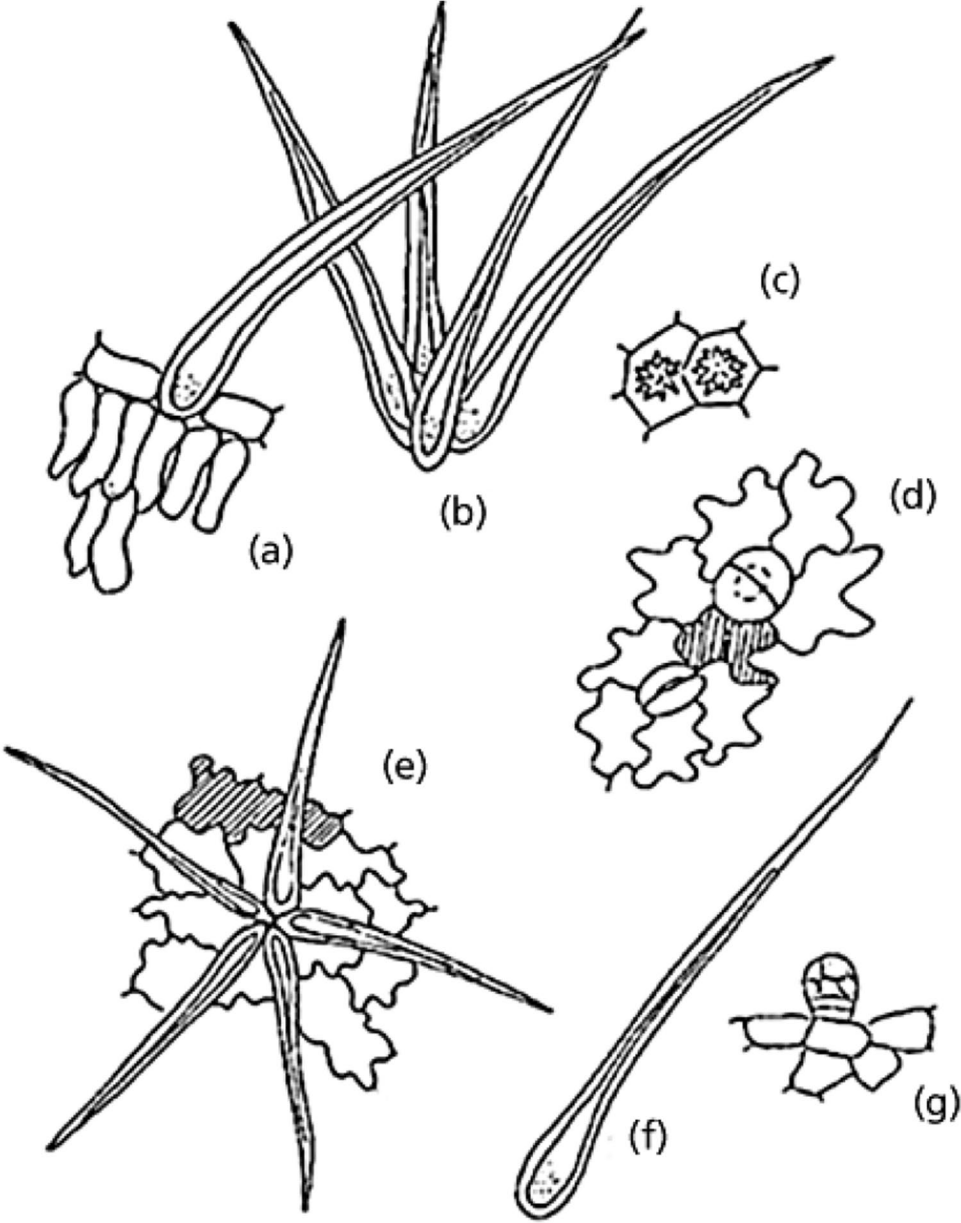
Morpho‐anatomical features of powdered leaf from *Malva sylvestris* L., *Malvaceae*. (**a**) Part of the palisade cells with non‐glandular trichome; (**b**) stellar trichome; (**c**) druses of calcium oxalate; (**d**) adaxial surface showing the stomata and mucilaginous cell; (**e**) abaxial surface with the stellar trichome and mucilaginous cell; (**f**) non‐glandular trichome; (**g**) detail of the epidermis with glandular trichome (Batistuzzo et al. 2002)

*Malva sylvestris* (*Malvaceae*) in traditional medicine dates back to around 3000 BC. Indeed, archaeological studies in Syria reveal some fossils of *Malva sylvestris* seeds in dental stones. This has been attributed by researchers in this field to the consumption of this plant as food but also because of its medicinal properties (Henry and Piperno 2008). This plant is characterised by simple, membranous, pubescent leaves that are velvety on both sides with long petioles. They have 3, 5, 7, or 9 shallow lobes and are palmar and orbicular to kidney-shaped in form. They have curved or sharp apexes, a tapered and razor-sharp underside, and a 7–15 cm diameter. Its venation is organised as an actinidrome with prominent and straight first-order veins, acute divergent for the second order and reticulate for the third. As for the last venation, it presents a marginal, incomplete form with veinlets and simple curves. The nipples, on the other hand, are large, polygonal in shape and show a frank development (Pljevljakušić et al. 2018; Batistuzzo et al. 2002) (Figs. 1 and 2).

### Agronomic, economic and ecologic aspects

Several studies report the weediness of *Malva* (Lavina et al. 1996; Zand et al. 2010) as it impedes the growth of many food plants except cereal crops, where no impact has been observed (Dutoit et al. 2007). *M. sylvestris* is a plant that can grow under different conditions, particularly on rocky soils, at varying pH and concentrations of nitrogen, phosphorus, and organic carbon (Godefroid et al. 2007). Its roots can firmly retain ions such as P, K, N and Mg when associated with tomatoes and beans during cultivation (Qasem 1992).

Scarification is the most effective method of germination, in addition to pollination by numerous insects that encourage the sustenance and propagation of *M. sylvestris* (Comba et al. 1999; Carreck and Williams 2002). The symbiotic relationship between *Malva* and many other organisms has been demonstrated. Indeed, Pappu et al. (2009) report in their work that *Malva sylvestris* represents the best host of *Aphis gossypii* compared to cotton and okra. Numerous microbes, including Cucumber Mosaic Virus, Cercospola malvcola, Tospovirus, Haritalodes derogatus, *Malva*pion *Malva*e, and Meloidogyne spp., might be added to this list (Pappu et al. 2009). Bees, butterflies, and hoverflies benefit from nectar from this plant’s nectar-secreting flowers (Comba et al. 1999). Treatment with herbicides can eliminate this plant, but this would negatively affect the economy and the environment (Qasem 1996; Jansen et al. 2000; Zand et al. 2010). Pinto et al. (2010) demonstrate the fungicidal capacities of methanolic extracts of *M. sylvestris* against *Colletotrichum lindemuthianum*, which is responsible for bean anthracnose. In the same vein, Madejón et al. (2006); Boojar and Goodarzi (2007) found that the roots of *Malva sylvestris* may help stabilise the soil by mitigating the harmful effects of copper by exclusion, as well as by regenerating degraded lands and copper-rich soils. In addition to this, Anastasakis et al. (2009) estimate that its mucilage may purify effluent by lowering turbidity by 96.3 to 97.4% in secondary effluent (12 mg/l of mucilage) and by 61 to 66% (62.5 mg/l of mucilage) in organic effluent.

Other research has also proven its ability to defend against ultraviolet radiation. Indeed, the leaves of *M. sylvestris* have the potential to transform the ozone contained in the apoplastic fluid surrounding the cells into superoxide radicals (O2—) in a brief period. This reactive oxygen formed around the veins produces apparent lesions that are then disseminated heterogeneously throughout the whole leaf surface, establishing this plant as a bio-indicator of ozone pollution. The plant’s susceptibility to ozone can have negative consequences for other crops in the nearby area, as it causes untimely senescence of the leaves, which results in a significant reduction in leaf growth and biomass, and in addition to seed quantity, flowering weight, and seedling growth, and hence plant development (Bergmann et al. 1995; Bender et al. 2006). On the other hand, the structural diversity of the rhizosphere’s main bacterial population is not appreciably affected by this prolonged exposure to the gas ozone (Dohrmann and Tebbe 2006).

### Applications in Traditional Medicine

Various studies have established the significance of *M. sylvestris* in traditional medicine. Many pharmacological studies have shown that *M. sylvestris* flower and leaf extracts can be used in treating various ailments, including digestive problems, dermatological diseases, menstrual pain, urological dysfunction, respiratory problems, gastrointestinal problems, abdominal pain and diarrhoea, respiratory infections, oral diseases (Leporatti and Corradi 2001; Cornara et al. 2009; Gasparetto et al. 2012a).

In many regions of the world, this plant is extensively utilised in traditional medicine (Tuttolomondo et al. 2014; Guarrera 2005). Elsagh et al. (2015) report in their clinical study the functional anticonstipative capacity of *Malva sylvestris*. Similarly, Conforti et al. (2008b); Sleiman and Daher (2009); Prudente et al. (2013b) and Prudente et al. (2017) report in their respective studies on the anti-inflammatory capacity to reduce topical inflammation of aqueous and hydroethanolic extracts of the aerial and leaf parts of *Malva sylvestris* in animal models. As established by some researchers, the composition of scopoletin, quercetin, and, in particular, malvidin 3-glucoside might account for the differences in anti-inflammatory activities.

According to Martins et al. (2014), the anti-inflammatory ability of *Malva* in vitro is connected to the amount of scopoletin, caffeic acid, and ferulic acid present in the plant’s composition. In addition, other authors (Idolo et al. 2010; El Beyrouthy et al. 2008; Pollio et al. 2008; Scherrer et al. 2005) have reported that compounds with anti-inflammatory properties in *Malva*, primarily those against gingivitis, abscesses, and dental pain, can be found in the plant’s leaves, flowers, and aerial parts (Idolo et al. In addition to this, Idolo et al. (2010), Cornara et al. (2009), and Lardos (2006) have shown the ability of leaves and blossoms to cure urological disorders, insect bites, burns, boils, and ulcerative wounds, among other conditions. In addition, Farina et al. (1995) proved the ability of a liquid extract of the flowers and leaves of *Malva sylvestris* to cure coughing and inflammation of the mucous membranes in treating cough and inflammation of the mucous membranes.

Modern literature attributes the high intake of *Malva* to its medicinal qualities, which include anti-ulcerogenic, antioxidant and anticancer characteristics, skin tissue integrity and anti-inflammatory properties (Quave et al. 2008). Ballero et al. (2001); Classen and Blaschek (2002) demonstrate in their studies that most of their therapeutic and pharmacological properties are attributed to the flowers and leaves because of their richness in flavonoids and mucilages.

It should be noted that *Malva sylvestris* is a medicinal plant consumed for its laxative, purifying, toning, and protective properties, particularly in the stomach (Guarrera 2003; Ishtiaq et al. 2007). It is most often consumed in the form of soup and salad. Its association with other medicinal plants potentiates the expected effect against certain ailments.

It (Malva sylvestris) has also demonstrated its phytotherapeutic and cosmetic potential in traditional medicine. Vitamin C, vitamin E, polyphenols, and -carotene, as well as other vital phytochemicals, might be the main driver behind its biological action (Barros et al. 2010). Gossypetin 3-sulfate-8-O—D-glucoside and hypocretin 3'-sulfate were found to be the primary flavonoid flavonoids in *Malva sylvestris* leaf tissue by Nawwar and Buddrus (1981), who also found that 70% of the extracts had higher inhibitory values than Amaranthus sp (Amaranthaceae), Sisymbrium thellungii O (Urticacea).

The tubers of Galium aparine L. teas. (Rubiaceae), Colocasia esculanta Schott (Araceae) and Aspalathus linearis (Burm.f.) R. Dahlgren (Fabaceae) (rooibos) and *Malva sylvestris* are also utilised in traditional medicine as safe food sources. According to Higashimoto et al. (1993), Sá Ferreira and Vargas (1999), and Schimmer et al. (1988), several herbs used in folk medicine may be poisonous, mutagenic or carcinogenic. Some other researchers have found secondary metabolites having a broad spectrum of biological properties in the polyphenolic compounds found in this plant, such as flavonoids, anthocyanins and tannins.

These compounds are beneficial in treating various diseases and conditions (Andújar et al. 2011; Manach et al. 2005; Shapiro et al. 2007). A study of the phytochemical composition of *Malva* reported that the aqueous fraction contained the most significant amount of phenolic compounds in total (3.39 mg/g), contradicting previous work (Barros et al. 2010; Conforti et al. 2008b). This difference is believed to be related to the dosing method applied, the growing conditions of the flat culture, and the growing location. Similarly, Samavati and Manoochehrizade (2013) also report that the polysaccharide extraction yield in this plant is higher than the yield under the optimal conditions suggested by Samavati.

### Veterinary uses

In addition to nutrition and application in human medicine, several studies reveal the importance of *Malva* in veterinary medicine. Indeed, Idolo et al. (2010) have shown that decoctions made from all parts of *Malva *and those boiled in oil were very effective in treating colic and cleaning rumens in cattle. In the same vein, Manganelli et al. (2001) have shown that *Malva* leaves applied in enema or compresses are highly effective against mastitis in cattle and swine constipation.

Additionally, according to Bonet and Valles (2007) and Akerreta et al. (2010), not only are infusions and extracts derived from the blooming aerial portions of this plant used as effective laxatives in horses, but they are also utilised as a therapy for wound infection, inflammation, respiratory diseases in horses, and enteritis and diarrhoea in cattle and swine, among other ailments. In the same sense, Bonet and Valles (2007) also report that *Malva* decoctions and infusions applied in the form of baths are used as a galactagogue in sows, antiseptics and against foot and mouth disease.

Applied by direct ingestion, leaf preparations have demonstrated laxative, anti-mastic and anti-ruminal methane production properties (Manganelli et al. 2001; González et al. 2010; Guarrera 2005). According to the authors (Viegi et al. 2003), *Malva* ointment can drain cattle abscesses and act as a potent cure for skin problems and reproductive and neurological disorders.

### Chemical composition

In addition to compounds whose properties have been previously demonstrated, other compounds, such as 8-hydroxyflavonoids of chemotaxonomic importance to *Malvaceae*, have been reported on *Malva sylvestris* leaves (Billeter et al. 1991).

According to Barros et al. (2010) on the nutraceuticals flavonoids, carotenoids, ascorbic acid, phenols, tocopherols, sugar and fatty acid compositions, as well as the antioxidant characteristics of *Malva sylvestris* plant various parts (flowers, leaves, immature fruits, and stems of leafy flowers), the *Malva sylvestris* plant might be considered a functional food or pharmaceutical.

Other phytochemical investigations, including those by Guil et al. (1996); Blunden et al. (2001); Cutillo et al. (2006); Hiçsönmez et al. (2009); Barros et al. (2010); Arabaci and Usluoglu (2012); Samavati and Manoochehrizade (2013), discovered that the various parts of mallow contain terpenoids, of phenolics derivatives, flavonoids, polysaccharides, chemical elements, mucilages, vitamins C, E, coumarins, beta-carotene, fatty acids, in particular essential fatty acids like various sterols, amino acids, omega-3 and omega-6, and enzymes including sulphite oxidase and catalase.

The techniques employed in their phytochemical analysis were gas chromatography-mass spectrometry (GC–MS), liquid chromatography-mass spectrometry (LC–MS), nuclear magnetic resonance (NMR), DPPH (2,2-diphenyl-1-picrylhydrazyl) scavenging activity, ferric reducing ability of plasma FRAP assays, and Fourier transform infrared spectroscopy (FT-IR). These techniques are commonly used to identify and quantify the various phytochemicals in a plant sample.

Gas chromatography–mass spectrometry is a common technique to identify and quantify small molecules in complex mixtures. In contrast, liquid chromatography-mass spectrometry is used to identify and quantify small molecules in complex mixtures. Nuclear magnetic resonance can be used to identify and quantify various organic compounds, including those found in plants (Awwad et al. 2015) (Farag et al. 2012). The DPPH scavenging activity and FRAP assays are common techniques used to assess the antioxidant activity of a plant sample (Delfine et al. 2017). Fourier transform infrared spectroscopy can be used to identify functional groups in organic molecules, including those found in plants (Awwad et al. 2015).

For isolated compounds, column-chromatography can be used as a purification step prior to identification by one of the techniques mentioned above (Bajpai et al. 2016). The column-chromatography can be performed using various materials, including silica, alumina, and carbon. Preparing the sample, packing the column, loading the sample, eluting the fractions, and analysing each fraction using thin-layer chromatography are all steps involved in isolating bioactive chemicals using column chromatography. High-performance liquid chromatography (HPLC) and nuclear magnetic resonance (NMR) spectrum analysis may be used to purify substances further; however, the methods most appropriate for a given study may vary (Bajpai et al. 2016).

### Amino acid/protein derivatives

Studies conducted by Classen and Blaschek (2002) on callouses cell cultures indicate that this plant is extremely rich (12.8% dry weight) in arabinogalactan protein (AGP) with a high proportion of mannose (3.5 mol%), glucuronic acid (3.2 mol%), galactose (57. 6 mol%), glucose (2.5 mol%), xylose (1.8 mol%), arabinose (31.0 mol%), and rham (0.4 mol%). In addition to these glycoproteins, amino acids such as alanine, arginine, glutamine, threonine, asparagine, hydroxyproline, and serine have been detected by HPLC chromatographic examinations. According to Classen and Blaschek (2002), hydroxyproline makes up about 0.8% of the total amino acid makeup. In the same line, authors such as Blunden et al. (2001) found the presence of trigonellin (1.9%, 1.0%, and 0.05%) and glycine betaine, which were found in the leaves at a concentration of 0.07%, flowers at 0.002%, and roots at 0.002%, based on their dry weight.

### Flavonoids

Only a few studies have discovered flavonoids in *Malvaceae* plants. There is some evidence to imply that, according to Sikorska and Matlawska (2004), *Malva sylvestris* may have an extra Functional group (OH) at the C-8 A ring and/or the C-5' B ring sites ß who reported that *Malva sylvestris* was saturated with flavonols and flavones. It was discovered that *M. sylvestris* leaves, flowers, immature fruits and flowering stem extracts’ total flavonoid content ranged from 210.8 to 46.6 mg per gram. Gossypetin 3-sulphate 8-O—d-glucoside (gossypine) and hypolaetin 3′-sulphate are the most abundant flavonoids in the leaves, according to Nawar et al. (1977) and Nawwar and Buddrus (1981).

**Fig. 3:**
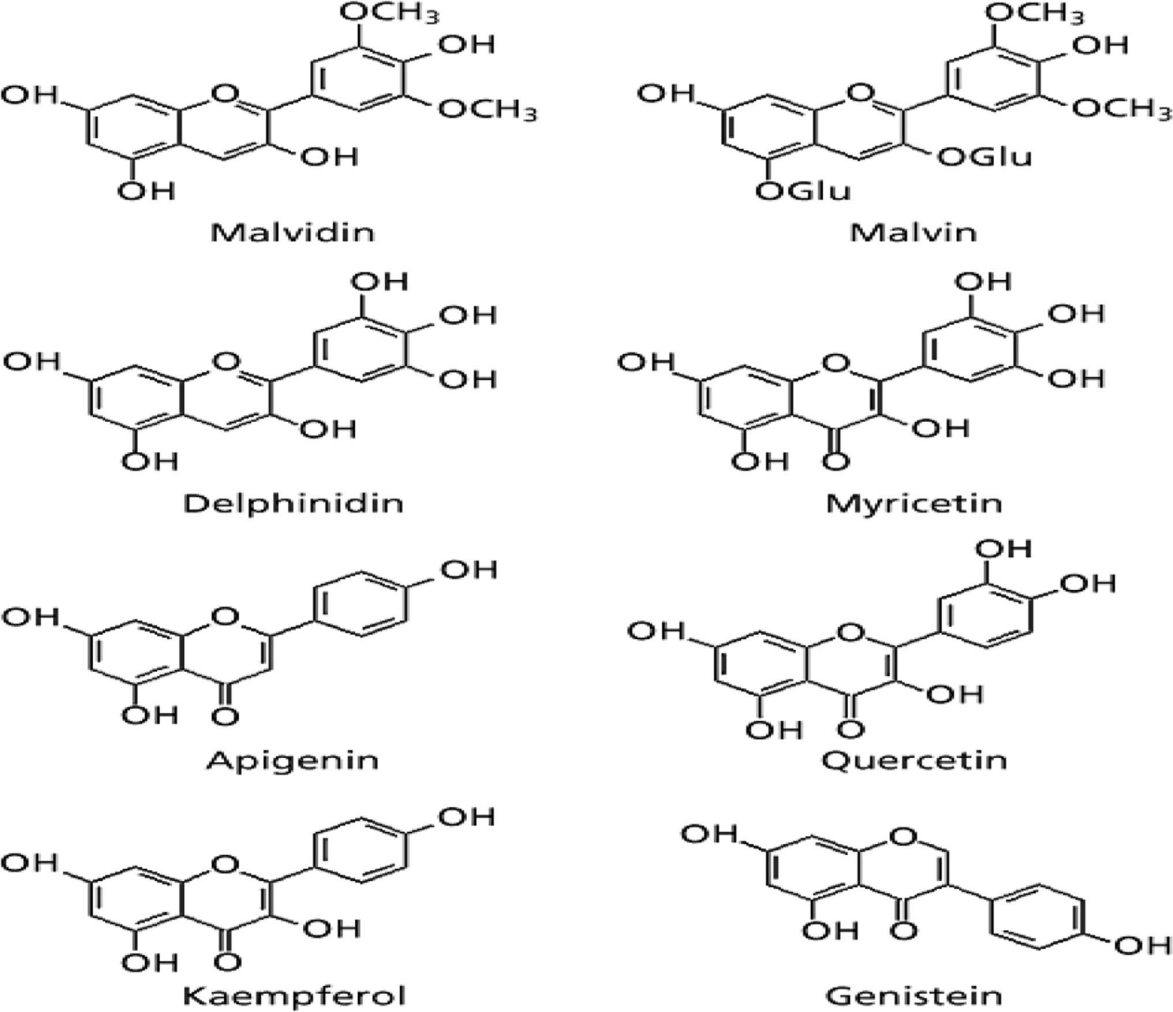
The chemical structures of selected flavonoids found in Malvaceae plants

Anthocyanins, particularly malvidin 3,5-diglucoside (malvin), are cationic flavonoids found in flowers (Brouillard, 1983; Pourrat et al. 1990). Delphinidine 3-O-glucoside and malvidin 3-O-(6′′-Malonylglucoside) are other antocyans discovered in flowers. Myricetin, apigenin, apigenin chloride, delphinidin and malvidin chloride are also included (a precursor to quercetin). About a third of all chemicals in the *Malva sylvestris* plant is made up of minor concentrations of other anthocyanins, including leucoanthcyanins, cyanidins, and petunidins, which have been identified (Pourrat et al. 1990) (Fig. 3).

### Mucilages

*Malva*les are the order of dicotyledonous plants with the most mucilage. Indeed, studies carried out for more than 50 years have revealed the presence of polysaccharides in the family *Malvaceae* (Franz 1966). Mucilages are compounds endowed with several properties, including anticomplementary and cough suppression. (Nelly et al. 2008; Tomoda et al. 1989). It is present in different amounts throughout the skin, with the highest concentrations in the specialised epidermal cells and the lowest concentrations in the mucilage idioblasts and channels (Classen et al. 1998).

Most of these chemicals are present in leaves, flowers, and roots respectively (Karawya et al. 1971; Hiçsönmez et al. 2009). In addition to sugars, galactose, fructose and glucose, as well as their acid derivatives, glucuronic and galacturonic acids are found in this part of the molecule, as well as uronic galacturonic acids. The remaining sugars and their acid derivatives include fucose; arabinose; mannose; raffinose; xylose; and 2′′-O-(4-O methyl—d-glucuronosyl)-xylotriose (Hiçsönmez et al. 2009; Classen and Blaschek 1998).

### Terpenoids

They mainly regurgitate monoterpenes, diterpenes, sesquiterpenes and nor-terpenes as terpenoids. Studies conducted on aqueous extracts of the leaves reveal the presence of linalol, linalol-1-oic acid, blumenol A, (6R,7E,9S)-9-hydroxy-4,7-megastigmadien-3-one, (3S,5R,6S,7E,9R)-5,6-epoxy-3,9-dihydroxy-7-megastigmene, (3R,7E)-3-hydroxy-5,7-megastigmadien-9-one, ( +)-dehydrovomifoliol, (3S,5R,6R,7E,9R)-3,5,6, 9-tetrahydroxy-7-megastigmene and (6E,8S,10E,14R)-3,7,11,15-tetramethylhexadeca-1,6,10-triene-3,8,14,15-tetraol (Cutillo et al, 2006).

**Fig. 4:**
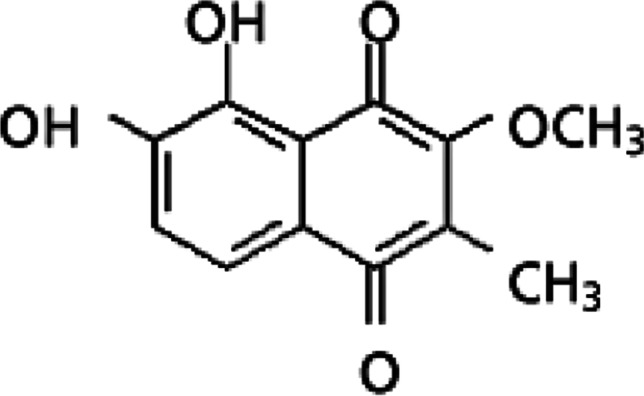
The chemical structure of malvone A (Gasparetto et al. 2012b)

In contrast, the oil extract from this plant seed is essentially full of terpineol, unlike the leaves, immature fruits, and flowers which are essentially made up of tetraterpenoids, which are carotenoids (Barros et al*.*., 2010). Among these terpenoids, it appears that *Malva* *sylvestris* isolated malvone A (2-methyl-3-methoxy-5,6-dihydroxy-1,4-naphthoquinone) is a potent antimicrobial, particularly against *Verticillium dahliae* (Veshkurova et al. 2006) (Fig. 4).

### Phenol derivatives

Phenolic composition investigations have revealed that *Malva* is a plant with high levels of phenolic compound intake in its leaves at 386.5 mg/g, blooming stems at 317.0 mg/g, flowers at 258.7 mg/g, and immature fruits at 56.8 mg/g. Cutillo et al. (2006) were the first to describe these phenolic compounds’ existence, characterisation and identification, despite a high concentration. These findings show the presence of 4-hydroxy-3-methoxybenzoic acid, 2-hydroxybenzoic acid, 4-methoxybenzoic acid, 4-hydroxy-2-methoxybenzoic acid, ferulic acid and tyrosol, 4-hydroxybenzyl alcohol, 4-hydroxydihydrocinnamic acid, 4-hydroxybenzoic acid, 4-hydroxy-3-methoxydihydrocinnamic acid, 4-hydroxycinnamic acid.

### Enzymes

Studying the enzymes in this plant has shown that it can degrade sulfur-containing amino acids by oxidative degradation. It should be noted that the absence of this enzyme in an individual can lead to death. It is mainly found in various animals and bacteria (Gasparetto et al. 2012b).

### Coumarins

Studies by Gasparetto et al. (2012b) discovered scopoletin (7-hydroxy-6-methoxycoumarin) and 5,7-dimethoxycoumarin, which has anti-cancer properties, in *M. sylvestris* leaves.

### Vitamins

There are two main groups of vitamins: water-soluble and fat-soluble. Among these, some, like tocopherols (vitamin E) and vitamin C, have antioxidant properties. These different compounds isolated from *Malva sylvestris* would thus be the origin of its antioxidant power. When it comes to tocopherols, they are divided into four primary classes (α, β, γ and δ), with α-tocopherol being the most important primary class in *Malva* (green plant tissues). As a result of its absorption and selective distribution in humans, It is the most powerful of the tocopherols in terms of antioxidant activity.

According to studies based on the measurement of these chemicals, large amounts are found in the leaves (106.5 mg), flowers (17.4 mg), flowering stems (34.9 mg), and immature fruits (100 mg) (2.6 mg). In addition, it shows that the same components are rich in vitamin C in high percentages, with the most significant amounts found in flowers (1.11 mg/g), immature fruits (0.27 mg/g), flowering stems (0.20 mg/g), and leaves (0.17 mg/g) (Barros et al. 2010). These findings show that Malva sylvestris is a potent antioxidant against reactive oxygen species (Gasparetto et al. 2012b).

### Fatty acids/sterols

Studies by Conforti et al. (2008a) show that *Malva* is a plant with many sterols, such as the steroids campesterol, stigmasterol and γ-sitosterol. Chromatographic studies (CPG) conducted on its oil reveal that it consists mainly of palmitic (26.6%), oleic (23%), *Malva*lic (11%), lauric (15.6%), myristic (6.6%), stearic (5.6%), palmitoleic (5.6%), linoleic (4%), vernolic (1.6%) and trace stearic acids (Gasparetto et al. 2012b).

Gasparetto et al. (2012b) also report that this variation in qualitative and quantitative fatty acid composition is closely related to growing conditions. Other studies report that in addition to seeds, flowers, leaves, immature fruits, and flowering stems are also sources of lipids.

Several fatty acids are found, ranging from saturated to unsaturated, short to long chains, and even odd. Many acids are present in this class, including caprylic, myristic, pentadecanoic, lauric, myristoleic, caproic, palmitic, palmitoleic, heptadecanoic, and stearic acids, as well as oleic, linoleic, -linolenic, arachidic, eicosenoic, cis-11,14-eicosadienoi, among others. Guil et al. (1996) report in their studies that extracts from *Malva* leaves treated with methanol and acetyl chloride has a lipid content of 0.47%, with α-linolenic acid as the majority lipid (42.2%). Its composition is essential, and essential fatty acids of the class of omega-3 and omega-6 class make this plant a nutraceutical food. Barros et al. (2010) have shown that omega-3 fatty acid-rich meals may help prevent diabetes, cancer, and cardiomyopathy, among other disorders.

### Radioecological compounds

The relationship between soil and plant has been demonstrated in the absorption rate of radioecological elements such as ruthenium-106. The uptake of this element (ruthenium-106) is highly dependent on the lineage (phylogeny) of the plant. The work of Willey and Fawcett (2006) reports the activity of 6570 Bq/g 103/106Ru in *Malva sylvestris*. In addition to this element, *M. sylvestris* also abounds in strontium (Sr) and technetium (Willey et al. 2010).

### Pigments

The presence of chlorophyll A, chlorophyll B, and xanthophylls in *M. sylvestris* has been reported in chromatographic investigations conducted on paper (Redzić et al. 2005).

### Chemical elements

The determination of the content of halogens, non-metallic compounds, and essential and non-essential metallic compounds in the leaves of *M. sylvestris* was carried out. In light of the findings of this experiment, which was carried out using inductively coupled plasma optical emission spectrometry (ICP-OES), it appears that Al, B, Ba, Bi, Ca, Co, Cr, Cu, Fe, K, La, Mg, Mn, Na, Ni, Pb, Si, Sn, Sr, Tl, U, Zn, Zr are the minerals that have been identified (Hiçsönmez et al. 2009). Another analysis, but this time quantitative by polarised X-ray fluorescence spectrometry, revealed the presence (mg/kg) of K (27,153.0), Mg (3340.0), Ca (19,477.4), Fe (2198.5), Al (1953). 9), Mn (99.6), Sr (52.1), Zn (40.8), Cu (16.7), Rb (12.0), Cr (10.4), Ni (8. 4), Co (4.1), Pb (1.5), Sn (1.1), Cd (0.6), Hg (< 0.1), S (5140.9), Cl (4971.4), P (3468.3), Br (51.7), I (6.1) and As (< 1.0). From work carried out by Khan et al. (2010), it appears that *Malva*’s heavy metal content (Cd, Cu, Ni, Pb and Zn) is significantly influenced by the soil composition of these substances. Awareness of the populations in these risk areas about the danger of ingestion of these metals is required.

### Biological activity

Studies conducted on the pharmacological properties of *M. sylvestris* have revealed its strong biological potential, as demonstrated by the patents obtained on this plant. Antimicrobial and anti-inflammatory studies evaluated various extracts and preparations of *Malva sylvestris* have led to its being recommended to treat several conditions like oral diseases.

#### 1- Antimicrobial activity

Maximum inhibitory dilution tests showed mouthwashes containing cetylpyridinium chloride base (CPC)-*M. sylvestris* extract combination had significantly higher antimicrobial capabilities than those containing CPC alone. Compared to CPC-only mouthwashes, this combination was potent against 28 different Staphylococcus aureus strains, whereas CPC-alone mouthwashes were only potent against three strains (Watanabe et al. 2008).

*Malva sylvestris* stem ethanol extracts showed antibacterial activity against methicillin-resistant *S. aureus strains*, according to Quave et al. (2008). They conducted planktonic growth and biofilm formation/adhesion assays on ethanol extracts from *M. sylvestris* stems.

According to the researchers, the half-maximum inhibitory concentration (IC50) of these extracts against *S. aureus* (IC50) was 32 g/ml in biofilm formation tests and had modest bacteriostatic effects in planktonic growth tests. This plant’s leaves and inflorescences were used to make ethanol extracts that exhibited modest antibacterial activity against Helicobacter pylori strains. On *H. pylori* 26,695 and *H. pylori* J99 strains, the hydroalcoholic extract had inhibitory diameters of 8 and 10 mm. According to Cogo et al. (2010), *Malva* inhibitory concentrations varied from 0.625 to 5.0 mg/ml in their lowest inhibitory concentration assays.

The lyophilised crude and methanolic extracts of Bonjar (2004), (De Souza et al. (2004), and others showed in their studies of inhibition diameter determination and agar diffusion tests that they had low inhibitory capacity on strains of *Escherichia coli, Pseudomonas fluorescens, Klebsiella pneumoniae, Pseudomonas aeruginosa, Serratia marcescens, Staphylococcus aureus, Aspergillus niger, Aspergillus candidus,* and *Penicillium spp*. were all inhibited by 0.60 g/ml of aqueous extracts from the leaves, but *Candida albicans* was completely ineffective in the presence of the extract (Magro et al. 2006).

#### Hepatoprotective effects

Several studies have also proved this plant’s antioxidant and free radical chelating abilities. When the systemic expression of reactive oxygen species is greater than the system’s capacity to detoxify or repair the damage, the term “oxidative stress” is used to describe this imbalance. Oxidative stress alters the organism’s redox state, resulting in the generation of peroxides and free radicals that damage all cellular components, including proteins, lipids, and DNA. Certain substances sensitive to oxidation reactions are involved in the cell signalling process as messengers. Their activities are interrupted after oxidation, which may lead to irreversible consequences on the signalling process, such as cellular damage.

Extracts of the flowers and leaves of *M. sylvestris* have been found to primarily consist of polyphenols, vitamins C, E, and beta-carotene; some polysaccharides and essential fatty acids, particularly omega-3 and omega-6, both of which have antioxidant properties; and some polysaccharides and essential fatty acids. It has been reported in the scientific literature that these components are present in significant amounts. Antioxidant capabilities were shown to be closely connected to the phenolic content of these plants (Javanmardi et al. 2003). Omega-3 has been shown to have antioxidant and preventive properties against kidney IR damage, according to Ashtiyani et al. (2012). Catalase, an enzyme that may neutralise H2O2 and protect against oxidative stress, is also included in this supplement (Arabaci and Usluoglu 2012). The kidneys were also safeguarded against vanadium-induced oxidative damage by a decoction of mallow (Marouane et al. 2011).

When determining the total antioxidant activity of natural compounds, the DPPH free radical scavenging test and the NBT reduction assay are the methods that are used most often. According to Gülçin et al. (2005), a decoction of *Malva sylvestris* has a significant amount of DPPH radical scavenging activity (IC50 = 0.68 g of dry *Malva sylvestris*/l), which the authors attributed to the plant's ability to donate hydrogen. Antioxidant compounds may also be tested for their DPPH-scavenging abilities, according to the researchers (Gülçin et al. 2005). In a research based on aerial portions of *Malva sylvestris*, Italian scientists DellaGreca et al. (2009) revealed that the extract plant at a concentration of 20 mg/l displays 24% DPPH scavenging activity.

At 5 g/l, mallows from Turkey showed a 46.19% scavenging effect on hydrogen peroxide (Nehir El and Karakaya 2004). Gossypium arboreum, for example, showed antioxidant capabilities, with an IC50 of 35.7 mg/l on the quantitative DPPH test, and the NBT reduction test was positive, according to Annan and Houghton (2008). *Malva sylvestris* decoction was shown to have an IC50 = 1 g of dry *Malva sylvestris*/l inhibitory effect on superoxide anion radicals. This information points to the likelihood that the antioxidant activity of *Malva sylvestris* may be attributable to compounds, most notably flavonoids, that are capable of scavenging superoxide and hydroxyl radicals via the process of single-electron transfer (Choi et al. 2002). On the other hand, Yazdanparast et al. (2008) revealed that antioxidant defence mechanisms developed alongside aerobic metabolism to combat ROS-induced oxidative damage. Furthermore, several metals, including vanadium, have been shown by Shi et al. (2004) to cause an increase in ROS, which in turn causes oxidative stress in cells. The buildup of thiobarbituric acid reactive substances (TBARS) in lipid peroxidation is now a crucial symptom of oxidative stress (Schaich, 1992).

The primary flavonols identified in *M. sylvestris* and *S. nigra,* respectively, were Kaempferol 3-O-rutinoside (0.84 mg/gdw) and quercetin 3-O-rutinoside (14.9 mg/gdw), and their antioxidant activity makes them suitable for use in extracts intended to promote health or in functional beverages or products (Zakhireh et al. 2013). The ethnobotanical and scientific properties of *M. sylvestris* have been examined in depth by Gasparetto et al. (2012b), who found that the plant may have therapeutic use. Similarly, Zakhireh et al. (2013) have noted that the clinical and toxicological aspects of its usage should be studied further.

The TBARS levels in the kidneys of vanadium-treated rats increased on day 30 compared to the control rats in our investigation. Other researchers found a spontaneous rise in TBARS in the kidneys of rats given vanadium. Studies in rats showed that exogenous Fe2 + enhanced kidney TBARS formation when animals drank water containing vanadium over four weeks (Scibior et al. 2006).

SOD, CAT, GPX, and other enzymatic and non-enzymatic defence mechanisms have been shown to limit ROS’s biological impacts, according to the research by Marouane et al. (2011) on ROS’s physical effects. Free radical scavenging may cause *Malvaceae* species' antioxidant characteristics, as previously suggested by Oboh and Rocha (2008). In the same line, Ilavarasan et al. (2003) found that treatment with Thespesia populnea, another *Malvaceae* plant, restored CAT and SOD activity to normal levels. Free hydroxyls in flavonoids and other phenolic compounds are most likely responsible for these antioxidant abilities. It is commonly accepted that plant extracts rich in polyphenols and flavonoids have antioxidant qualities and health benefits (Elingold et al. 2008). For the most part, the positive effects of *Malva*-based preparation (decoction or infusion) may be traced back to the high concentration of these substances.

There is little doubt that flavonoids significantly impact the oxidation of fats, as shown by Lin et al. (1998) and Sánchez-Moreno et al. (1999). In addition, these antioxidant properties have been tested in various ways. Using aqueous extract at doses of 20 and 100 g/ml, some researchers found a decrease in 1,1-diphenyl-2-picrylhydrazyl (DPPH) test scavenging activity between 24 and 30%. When tested, this same aqueous carotene extract at 0.1 mg/ml showed an 87% antioxidant effect on linoleic acid. The exact quantity of *M. sylvestris* essential oil was shown to have significant antioxidant activity by Ak and Gülçin (2008) and Ferreira et al. (2006). The antioxidant activity of leaf and aerial ethanolic extracts, n-hexane, dichloromethane, and seed methanolic extracts was moderate to low (Conforti et al. 2008b; Nehir El and Karakaya 2004; Kumarasamy et al. 2007).

The antioxidant activity of methanolic extracts obtained from leaves, flowers, immature fruits, and flowering stems was investigated by Barros et al. (2010) using several different assays and models, including absorption of DPPH radicals, neutralisation of linoleate free radicals, the malondialdehyde-thiobarbituric acid (MDATBARS) complex and carotene models. The DPPH experiment revealed that all parts of the plant, except immature fruits, have antioxidant potential (EC50 value of 0.6 mg/ml). The green fruits had an EC50 value of 4.47 mg/ml. This includes powerful antioxidant properties obtained on methanolic extracts from the leaves, which include the ability to scavenge free radicals, the ability to reduce them, in addition to having the capacity to reduce lipid peroxidation in liposomes (EC50 = 0.04 mg/ml) and brain cell homogenates (EC50 = 0.09 mg/ml), among other properties.

In this portion of the plant, antioxidants like phenols, flavonoids, carotenoids, and tocopherols are responsible for the leaf methanolic extracts’ potent antioxidative activities. Studies comparing plants from various European and Asian countries found that methanolic extracts from plants collected in northeastern Portugal had the highest antioxidant activity. These studies also showed the impact of geographical area and climatic conditions on the level of antioxidant properties that plants possess (Barros et al. 2010).

This medicinal plant also has analgesic characteristics, according to Esteves et al. (2009), who found that an aqueous lyophilised extract from *M. sylvestris* leaves significantly reduced pain in rats. Acetic acid-induced abdominal constrictions were decreased by 76.4% when the section was injected intraperitoneally (10 mg/kg) during the abdominal constriction test. The neurogenic phase of a formalin test, induced by formalin injection, resulted in a 61.8% drop in excessive licking. In contrast, the inflammatory phase resulted in a 46.6% drop in excessive licking. On the other hand, another finding was that capsaicin resulted in a 62.9% reduction in the total time the animal was licking the injected paw. The only pain model used was the hotplate model; nevertheless, the aqueous extract did not seem to have any positive benefits in any research that investigated it. Antinociceptive effects may be attributable to the aqueous extract's ability to block the cyclooxygenase pathway rather than its ability to stimulate the opioid receptors (Esteves et al. 2009).

### Complementary and alternative medicines; including the use of plants

For thousands of years, people throughout the globe have acknowledged the usefulness of plants in medicine, whether it be contemporary or traditional (Brown 1980; Chevallier 1996; Barnes et al. 2008). It is reported that over 100 million Europeans utilise complementary and alternative medicine (CAM), including phytotherapy, to treat diseases for which modern medicine has no cure (Busuttil-Griffin et al. 2015). (Organization 2013; Busuttil-Griffin et al. 2015). Most people in developed countries like Australia and North America, as well as those in low and middle-income countries like Asia and Africa, are still relying on conventional medicine for the majority of their healthcare requirements (Organization 2013, 2004; Weiner and Ernst 2004; Barnes et al. 2008). Since at least the eighth century BCE, the young leaves and shoots of *Malva sylvestris* have been used as a therapeutic supplement (Chevallier 1996; Barros et al. 2010). For centuries, *Malva sylvestris* was renowned as a “cure-all” and utilised for its relaxing effects, as Busuttil-Griffin et al. (2015).

One of the most often used medicinal plants in Malta was known as “il-Ħubbejża” or “Ħobbejża” (Lanfranco 1975) and “purifying the blood” after boiling (Lanfranco 1992).

Damaged tissues are restored to their pre-injury condition via the wound-healing process, whereas wound contraction is the process through which the wound area shrinks (Nayak et al. 2007). Wound healing is mainly influenced by the patient’s overall health, the tissue’s capacity to mend, and the severity of the injury.

The literature does not mention *M. sylvestris* as a therapy for this disease, apart from its traditional usage in the wound healing process in Iran. Detailed ethnobotanical studies (Pirbalouti, Ghorbani and Zargari 1990) reveal that this plant is widely used in Iran to treat infectious, inflammatory and microbial skin diseases and cure wounds.

Phillips et al. (1991) found that wound healing involves a multi-factor sequence of events involving several cellular and metabolic reactions. During this process, the skin’s anatomical continuity and functional state are reconstructed and regenerated (Phillips et al. 1991). Many studies have shown that *M. sylvestris* application speeds up wound healing and repair. One such study showed that an ordered epidermis covered the total thickness of the wound region with mature scar tissue in the dermis. This skill became more apparent when the statistics were compared to those of the other groups. *M. sylvestris* considerably improved the rate of wound contraction and collagen turnover in histological evaluations. Biochemical markers for tissue collagen, such as collagen’s amino acid hydroxyproline, were discovered by Phillips et al. (1991). They found collagen to be the primary structural component supporting and strengthening extracellular tissue.

Several studies have shown that the flowers of the *M. sylvestris* plant can be used to treat wounds that have been cut or infected with bacteria. These include eczema, inflammatory diseases such as gastritis and bronchitis (SamavSamavati and Manoochehrizade 2013), rheumatism, acne and skin care. The effects of this plant on the body, such as its diuretic, laxative, laxative, spasmolytic, lenitive, choleretic, and antioxidant properties, are also explored. Researchers have identified many different compounds in *M. sylvestris*, such as polyphenols, vitamins C and E, beta-carotene (Barros et al. 2010b), anthocyanidines, naphthoquinones, flavonoids, and mucilaginous polysaccharides, in addition to tetrahydroxylated linear diterpene, monoterpenes, and phenol derivatives (Barros et al. 2010 Eleven different chemicals have been isolated from *M. sylvestris* through the use of the Cutillo and aqueous extraction methods. These chemicals include 4-hydroxybenzoic acid, methyl 2-hydroxydihydrocinnamate, scopoletin, and malvone A 2-methyl-3-methoxy-5, and 6-dihydroxy-1,4-naphthoquinone, as well as malvone B and malvone C. It was discovered by Gasparetto et al. (2012a) that the composition of *M. sylvestris* flowers and aerial parts possesses additional healing potential. These properties include anti-inflammatory, anticarcinogenic, and positive outcomes on gum disease and abscesses, tooth pain, and urological illness, in addition to positive effects on insect bites and ulcerous wounds.

### Laxative properties

The amount of plant matter consumed, the section of the plant used, and the method of preparation all influence whether *M. sylvestris* has a laxative impact on the intestines when swallowed (Brown 1980; Gasparetto et al. 2012b). *M. sylvestris* has been described as an intestinal stimulant and an effective laxative for young infants by Boulos (1983) and Lim (2014). According to the letter sent by Cicero (106–43 BCE), decoctions made from Mallow (*M. sylvestria*) and Chard (*B. vulgaris*) caused him to suffer from diarrhoea (Woodville 1810).

### Fibre crop

*M. sylvestris* is cultivated for the stem fibre it produces. It is a member of the *Malvaceae* family, which also contains plants such as Hibiscus cannabinus, Urena lobata, and Hibiscus sabdariffa (De Rougemont 1990). According to Detmers (1909), the high fibre content of *M. sylvestris* indicates that it may be classified as a bast fibre crop and, as a result, is not suited for cultivation. Some authors believe that the high mucilage content of *M. sylvestris* (Chevallier 1996; Arber 2002) may be a factor in the plant’s laxative activity. Mucilage is a component of dietary fibre (Committee 2001), and *M. sylvestris* is recognised for its high mucilage content (Chevallier 1996; Arber 2002). (Chevallier 1996; Arber 2002). (Kallas 2010).

### Fibre consumption

This is consistent with Sanders (1995), who pointed out the distinct differences in illness patterns in fibre-rich communities vs. those in low-fibre populations. Constipation, one of the disorders that was and still is linked to a diet low in fibre, is one of the results (DeVries 2003). According to Heaton’s study from 1997, treating constipation with plant fibre has a long history of success. The ability of fibres to absorb and retain water in the digestive tract is primarily due to the bulking and softening effects on the stool. As a result, the stool volume increases (Dwyer et al. 1978). Microbial fermentation produces short-chain fatty acids, which may be used to both prevent and cure constipation (Sanders 1995). (Eswaran et al. 2013). Consuming the proper quantity of fibre also helps regulate intestinal transit time, which helps to battle both diarrhoea and constipation and reduces the prevalence of bowel-related diseases, according to Slavin and Jacobs (2010). (Dwyer et al. 1978).

### Antiproliferative activity

Multiple studies have shown that the *M. sylvestris* plant has antiproliferative properties. Conforti et al. (2008a) examined the potential antiproliferative properties of ethanolic and hydroalcoholic leaf extracts of *M. sylvestris* in several human tumour cell lines. Researchers found that the plant's MTT (3(4,5-dimethylthiazol 2'yl)2,5-diphenyl tetrazolium bromide) considerably decreased the growth of B16 murine and A375 human melanoma cells (97% compared to the control) (58%). Sulphorhodamine B, on the other hand, did not affect the proliferation capability of MCF-7, LNCaP, C32, or renal adenocarcinoma cell lines when used. A different species of Microsporum, *M. sylvestris*, is associated with protecting the skin's mucosa and the integrity of the membrane. Cosmetics, topical chemicals, and moisturisers have all been developed due to their potential to enhance dermatological properties. According to their research, skin irritation was relieved, mucus production was increased, and free radicals were scavenged by Gasparetto et al. (2012b). *M. sylvestris* aqueous extracts and indigestible chemicals increase the structural integrity of human skin and tissues, according to Seiberg et al. (2006) and Seiberg et al. (2010). Because of their solid cytotoxic activity and antiproliferative properties, M. sylvestris solutions and lotions were used to treat alopecia and other capillary problems. As a result of its ethnomedicinal ability, it has been employed since ancient Greece and Rome for treating several ailments. In addition to its usage in pancakes and salads, cooked greens, and, in certain countries, stuffing, immature fruits may be eaten raw as a snack. It has also been used in a variety of other culinary applications (Dogan et al. 2004).

Sleiman and Daher (2009) found that aqueous extracts of M. sylvestris have anti-ulcerogenic effects in rats with generated stomach ulcers. Using a 500 mg/kg body weight dose for one month, they found a maximum of 37% protection. Anti-ulcerogenic action was compared to cimetidine, a reference medicine, and found to be equivalent to the 30% maximum level of protection.

### Sustainable synthesis of metal nanoparticles

Recent studies have highlighted the plant’s role in the biological production of silver nanoparticles because of its low cost and environmentally benign attributes. Metabolites from bacteria, mold, and plants are helpful in the green production of metal nanoparticles in many studies. Furthermore, silver nanoparticles may be produced by drying the silver in the shade and grinding it into a fine powder using an electric grinder (case of *M. sylvestris* leaves). To do this, a magnetic stirrer was used to combine 50 g of powdered dried leaves with 500 ml of water, which was brought to a boil and then allowed to cool. After letting the resultant suspension settle for 3 h, it was filtered using Whatman n. one filter paper, and the aqueous filtrate was kept at a temperature of 10 °C until it could be tested (Govindarajan et al. 2016).

### Synthesis of silver nanoparticles

**Fig. 5:**
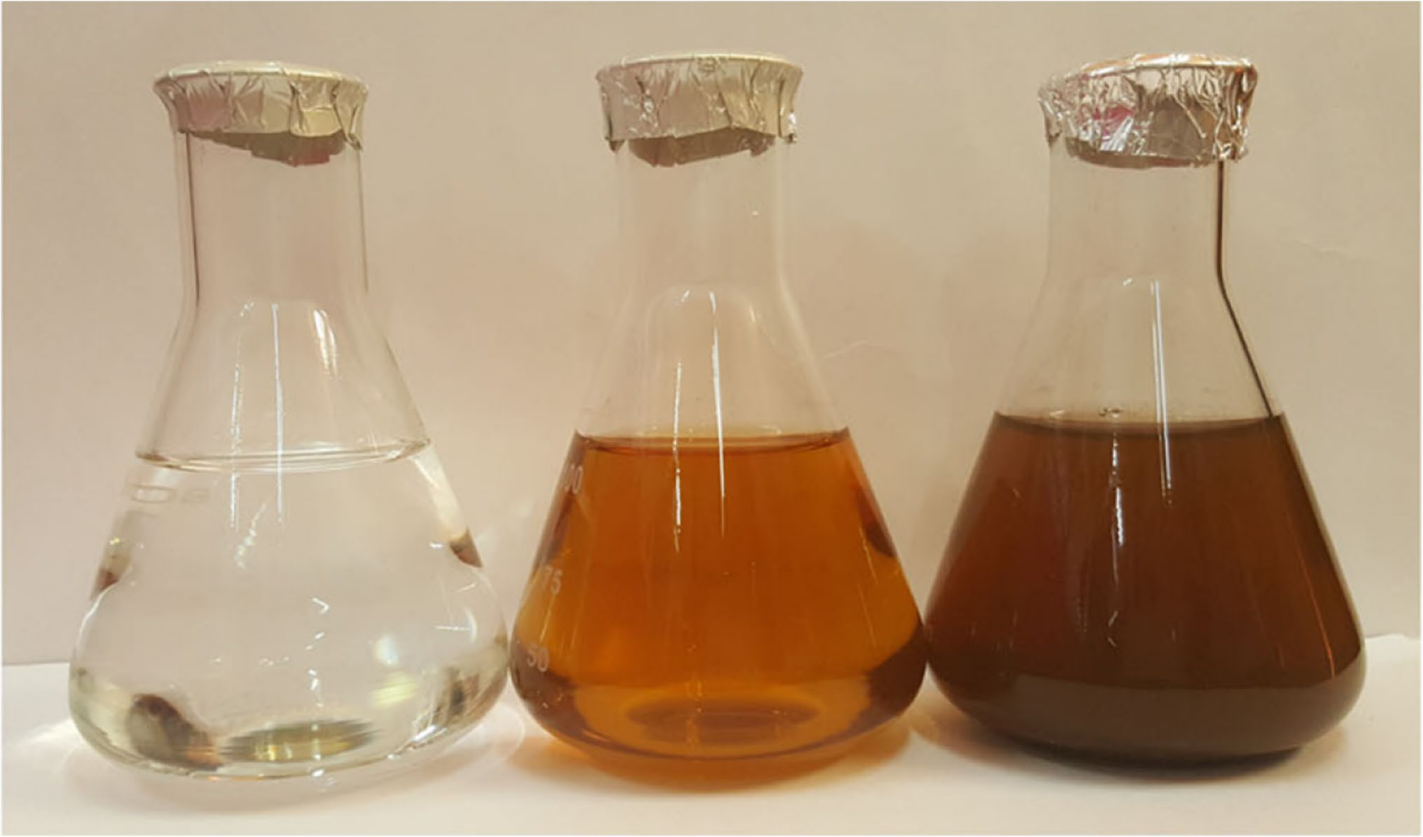
A comparison of the colour intensity of the aqueous extract of *Malva sylvestris* before and after reducing the amount of silver nitrate (1 mM). The change in hue shows that Ag + has been reduced to an elemental nanosilver (Govindarajan et al. 2016)

The leaves of the *M. sylvestris* plant were washed and then finely chopped before 300 mL of water was added. The mixture was heated for 5 min in an Erlenmeyer flask with 100 mL of sterilised double-distilled water before being decanted. After that, it was filtered using Whatman filter paper number 1 and stored at a temperature of − 15 °C until the colloidal extract it generated was evaluated. After being treated with aqueous one mM AgNO3, the filtrate was homogenised in a Milli-Q flask and then incubated at room temperature. This was done to produce a standardised solution (21.2 mg AgNO3 powder in 125 mL of Milli-Q water). Next, the leaf extract of *M. sylvestris* was used to decrease an aqueous solution of AgNP at room temperature for 10 min, and the brown-yellow solution formed, showing that AgNP had been developed from AgNO3 (Govindarajan et al. 2016) (Fig. 5).

### Synthesis of copper oxide nanoparticles

A typical reaction combination produces oxide nanoparticles using M. sylvestris, which are revealed after being made. In essence, 400 ml of 4 mM copper chloride dehydrate CuCl2,2H2O was combined with 10 ml of aqueous leaf extract of *Malva sylvestris. The* mixture was then agitated magnetically at room temperature until the colour changed from a light blue to a light green tint. This process took approximately 30 min. Later, the mixture is heated at 80 °C for 2 min before being treated with 1 M sodium hydroxide, which is dropped into the liquid in drops. To notice the production of water-soluble monodispersed copper oxide nanoparticles owing to the interaction between sodium hydroxide and copper ions as soon as possible, we observed the spontaneous change of green mixture to brown precipitate as soon as it was feasible.

**Fig. 6:**
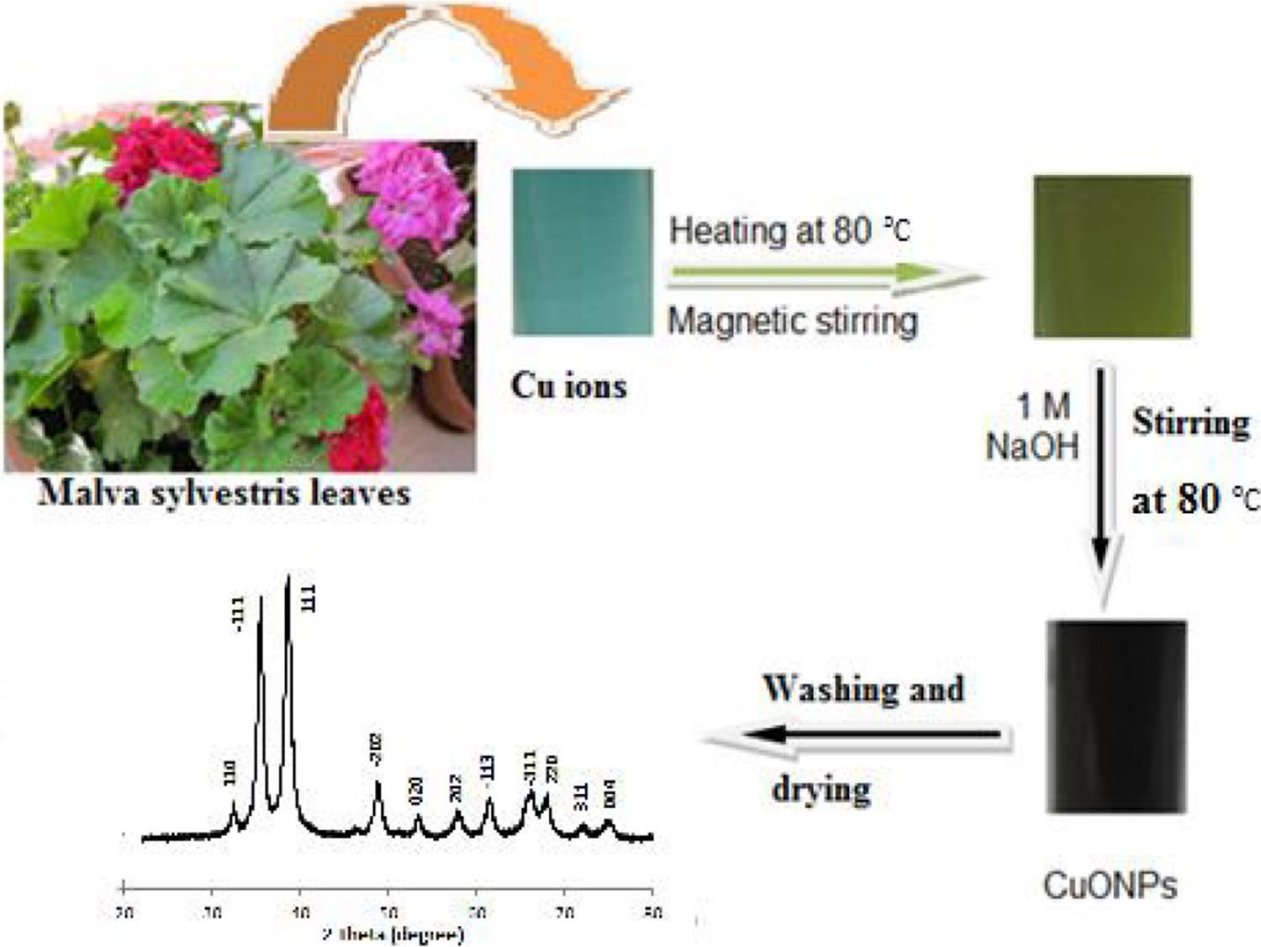
Copper oxide nanoparticles synthesised using a green method (Awwad et al. 2015)

In the trials, the levels of copper chloride solution and leaf extract were varied from 1 to 4 mm and 1–10% by volume, respectively. The brown precipitate was removed after the sergeant was removed and rinsed with deionised water many times before being cleaned with ethanol to eliminate any remaining contaminants before being used to make the finished product. The product was dried at 60 °C in a vacuum oven overnight, and the brown powder was obtained as the copper oxide nanoparticle (Awwad et al. 2015) (Fig. 6).

### Toxicity

The biochemical profiles of rats eating plant shoot aqueous leaf extract were examined by Sleiman and Daher (2009). They found that dosages of 400 and 800 mg/kg body weight substantially raised blood triglycerides but had no effect on glycaemia, liver enzyme activity, or lipid profiles. Aqueous extracts alone or combined with Cotinus coggygria extract have been shown to dramatically lower levels of triglycerides and urinary uric acid in humans, as Seiberg et al. (2006) and Seiberg et al. (2010). Even though it is frequently used in human food and traditional medicine, toxicological tests have shown that it may still have negative health consequences at large doses. Microtox acute toxicity of the hydroalcoholic extract was found to be incredibly near to the defined maximum in this instance, according to Gasparetto et al. (2012) (20% of bioluminescence inhibition). Because of this, further studies are needed to determine the toxicity of *M. sylvestris* extract.

## Conclusion

This review has presented an overview of the antinociceptive and anti-inflammatory activities and the cytotoxic effects of *Malva sylvestris* on different types of cancer cells. It has also summarised the work done on developing copper oxide nanoparticles using *Malva sylvestris* leaf extract and its potential use in food and medicine. The leaves and flowers of *Malva sylvestris* are the most commonly used parts of the plant and contain a variety of phytochemicals such as flavonoids, mucilages, terpenoids, phenol derivatives, coumarins, sterols, tannins, saponins, alkaloids. These phytochemicals are responsible for the many pharmacological activities of *Malva sylvestris,* such as anti-inflammatory, antimicrobial, hepatoprotective, laxative, antiproliferative and antioxidant properties. *Malva sylvestris* has also been shown to be safe when used in traditional medicine to treat various ailments such as cough, cold, diarrhoea, and constipation. Most studies have focused on the analgesic and anti-inflammatory effects of *Malva sylvestris* extracts, with fewer studies investigating other areas, such as diabetes and cancer. However, more clinical studies are needed to assess this plant's safety and efficacy in treating other conditions. Further research is also necessary to determine whether there are any toxicological consequences to consuming large doses of *Malva sylvestris* extracts or nanoparticles over a long period.

## Data Availability

Data sharing is not applicable to this article as no datasets were generated or analysed during the current study.
